# Monocarboxylate transporter 1 promotes classical microglial activation and pro-inflammatory effect via 6-phosphofructo-2-kinase/fructose-2, 6-biphosphatase 3

**DOI:** 10.1186/s12974-019-1648-4

**Published:** 2019-11-28

**Authors:** Liang Kong, Zehua Wang, Xiaohong Liang, Yue Wang, Lifen Gao, Chunhong Ma

**Affiliations:** 1 Key Laboratory for Experimental Teratology of Ministry of Education and Department of Immunology, School of Basic Medical Sciences, Shandong University, Jinan, Shandong 250012 People’s Republic of China; 2 Department of Neurobiology, Shandong Provincial Key Laboratory of Mental Disorders, School of Basic Medical Sciences, Shandong University, Jinan, Shandong People’s Republic of China; 3 Key Laboratory of Infection and Immunity of Shandong Province, School of Basic Medical Sciences, Shandong University, 44 Wenhua Xi Road, Jinan, Shandong 250012 People’s Republic of China

**Keywords:** Classical microglial polarization, MCT1, PFKFB3, Glycolysis, Lactate, Neuroinflammation

## Abstract

**Background:**

Microglia, the resident macrophages of central nervous system, have been initially categorized into two opposite phenotypes: classical activation related to pro-inflammatory responses and alternative activation corresponding with anti-inflammatory reactions and tissue remodeling. The correlation between metabolic pattern and microglial activation has been identified. However, little is known about the mechanism of metabolism-mediated microglia polarization and pro-inflammatory effect.

**Methods:**

Metabolic alteration was analyzed in different phenotypes of microglia in vitro. LPS-induced neuroinflammation and sickness behavior mouse model was used to investigate the effect of lactate on classical microglial activation in vivo.

**Results:**

Glycolysis-related regulators, monocarboxylate transporter 1 (MCT1), MCT4, and pro-glycolytic enzyme 6-phosphofructo-2-kinase/fructose-2, 6-biphosphatase 3 (PFKFB3), were specifically increased in LPS-stimulated primary microglia and microglia cell line BV2. Knockdown of MCT1 suppressed glycolysis rate and decreased LPS-induced expression of iNOS, interleukin-1β (IL-1β), IL-6, and phosphorylation of STAT1 in BV2 cells. Importantly, MCT1 promoted PFKFB3 expression via hypoxia-inducible factor-1α (Hif-1α), and overexpression of PFKFB3 restored the classical activation of BV2 cells suppressed by MCT1 silence. All above strongly suggested that MCT1/PFKFB3 might accelerate LPS-induced classical polarization of microglia probably by promoting glycolysis. Interestingly, additional administration of moderate lactate, which may block the transport function of MCT1, decreased LPS-induced classical activation and expression of PFKFB3 in BV2 cells. Intracerebroventricular injection of lactate ameliorated LPS-induced sickness behavior and classical polarization of microglia in mice.

**Conclusions:**

Our results demonstrate the key role of MCT1 in microglial classical activation and neuroinflammation in pathological conditions. In addition, lactate administration may be a potential therapy to suppress neuroinflammation by altering microglial polarization.

## Introduction

Microglia, the tissue-resident macrophages in the central nervous system (CNS), play important roles in phagocytosis, cell migration, antigen presentation, and cytokine production [1]. Accumulated evidences have demonstrated the critical role of microglia in neuroinflammation in multiple neurologic diseases, such as Alzheimer’s disease and ischemic stroke [1–5]. Similar to macrophages in peripheral tissues, microglial activation in the CNS is heterogeneous, which can be categorized into two opposite types: M1-like phenotype (classical activation) and M2-like phenotype (alternative activation). M1 like phenotype represents pro-inflammatory responses, including the release of pro-inflammatory cytokines, such as tumor necrosis factor-α (TNF-α), interleukin-1β (IL-1β), and reactive oxygen species (ROS). M2-like phenotype plays critical roles in tissue repair and neuroprotection, and exhibits anti-inflammatory reactions by expressing IL-10 and transforming growth factor-β (TGF-β) [6–9]. iNOS, TNF-α, IL-1β, IL-6, and STAT1 are important markers of M1 phenotype, while M2 markers mainly include Arg1 and CD206. Although the M1/M2 polarization is oversimplified to describe the heterogenecity of activated microglia, M1/M2 balance is helpful to understand the dual characters of microglia in neuroinflammation.

Increasing evidences support the critical role of multiple metabolic pathways in macrophage reprogramming [10, 11]. Rather than depending on mitochondrial ATP production, M1-like macrophages display high glycolysis rates, which can be induced by the expression of glucose transporter 1 (GLUT1) and pro-glycolytic 6-phosphofructo-2-kinase/fructose-2, 6-biphosphatase 3 (PFKFB3) [10, 12]. Monocarboxylate transporters (MCTs) are also important regulators in glycolysis by transporting redundant lactate outside of the cellular. Conversely, M2-like phenotype displays low glycolysis rates without expression of PFKFB3 but appears high rates of mitochondrial fatty acid oxidation and oxidative phosphorylation [10, 13, 14]. Actually, artificial reprogramming of macrophage polarization is somewhat successful in clinic [15]. Therefore, glycolysis-related regulators influencing macrophage/microglia polarization may benefit therapy for different inflammatory diseases including neuroinflammation such as ischemic stroke. Recent data have confirmed the link between metabolism and microglia polarization similar to traditional macrophages. Indeed, a strengthening of glycolysis is observed in M1-like microglia, which is dependent on the increase of hexokinase and lactate dehydrogenase activity, and high expression of GLUT1 [16, 17]. However, it is still unknown whether glycolysis alteration could directly regulate microglial polarization. Considering that glucose is the main energy source in CNS, it is necessary to fully understand the role of glucose metabolism in microglial polarization. MCTs, the main transporters of lactate, are regarded as the special step of anaerobic glycolysis but not TCA cycle and oxidative phosphorylation. Evidences reveal that MCT1 protein is increased in N11 microglial cells after stimulation with TNF-α [18]. However, the key role of MCTs and glycolytic metabolite lactate in microglial activation remains unclear.

In order to address the role of MCTs in microglial polarization and evaluate the potential role of microglia metabolic reprogramming in limiting its pro-inflammatory effect, lentivirus-mediated MCT silencing and overexpression of PFKFB3 were applied in LPS or IL-4-stimulated microglia. Besides, the open field test was used to investigate the effect of lactate on LPS-induced neuroinflammation and sickness behaviors in mice. Our data demonstrated the critical role of MCT1/PFKFB3-promoted glycolysis in classical microglia activation and pro-inflammatory effect.

## Materials and methods

### Animals

Male C57BL/6 mice (8 weeks old), weighting 25–30 g, were used in this study. Mice were housed at 22 °C on a 12-h light/dark cycle with abundant water and food. All procedures referred to the National Institutes of Health Guide for the Care and Use of Laboratory Animals and were approved by the Institutional Animal Care and Use Committees of Shandong University.

### Cell cultures and treatment

Primary microglial cells were obtained from the brain of C57BL/6 mouse pups postnatal days 1–3 [9, 19]. In short, the cerebral cortex without the hippocampus and striatum was digested with 0.25% trypsin and DNase for 15 min at 37 °C. Cells were filtered with 70-μm nylon mesh and seeded into T75 flask (Corning). Resultant cells were maintained with DMEM and 10% fetal bovine serum supplemented with 1% of a penicillin/streptomycin solution. After 2–3 weeks, microglia were removed from flask by shaking. BV2 microglial cells were cultured with DMEM and supplemented with 10% heat-inactivated fetal bovine serum, 1% of a penicillin/streptomycin solution, and 2 mM L-glutamine. All cells were kept at 37 °C under 5% CO_2_ atmosphere. Hif-1α inhibitor KC7F2 (40 μM, dissolved in dimethyl sulfoxide, MCE, HY-18777) and MCT1 substrate L-lactate (5 mM, dissolved in PBS, Sigma, L7022) or pyruvate (5 mM, dissolved in PBS, Sigma, P5280) were used in this study.

### RNA isolation and real-time quantitative PCR

After treatment, cells were used to extract RNA with TRIzol-A^+^ RNA isolation reagent (TIANGEN). The total RNA was applied to synthesized cDNA by the Revert Aid First Strand cDNA Synthesis Kit (Fermentas). All protocols were performed according to the manufacturer’s instructions. Quantitative RT-PCR was performed in a Cycler (Bio-Rad) using SYBR Green reagent (TIANGEN) to quantification. The *β-actin* mRNA levels were used to normalization according to 2^-ΔΔCT^ method. The primer sequences used were listed in Table 1.

**Table 1:** Sequence of primers used for quantitative real-time PCR

Symbol	GenBank	Forward primer (5′-3′)	Reverse primer (5′-3′)
iNOS	NM_001313921	GAGCGAGTTGTGGATTGTC	CTGCCTATCCGTCTCGTC
Arg1	NM_007482	ATCTGCCAAAGACATCGT	CATCACCTTGCCAATCCC
MCT1	NM_009196	GAGGTCCTATCAGCAGTATCTT	CCAGTGGTCGCTTCTTGT
MCT2	NM_011391	CACTGGCTCCTTTCAATC	CTGGCTTTCTTCAGAGTTG
MCT4	NM_001038653	ACTTCAACAAGCGTCGCCCTAT	TCAGTCCCTCCGCCTACCTG
PFKFB3	NM_001177752	AGCCTCTTGACCCTGATA	TTCTTGCCTCTGCTGGAC
IL-1β	NM_008361	ATTGTGGCTGTGGAGAAG	ATCTCGGAGCCTGTAGTG
IL-6	NM_001314054	TGCCTTCTTGGGACTGAT	TTGCCATTGCACAACTCTTT
TNF-α	NM_001278601	GGCGGTGCCTATGTCTCA	CCTCCACTTGGTGGTTTGT
Hif-1α	NM_001313919	CATCATCTCTCTGGATTTTG	GAAGAGGGAAACATTACATC
β-actin	NM_007393	CGTTGACATCCGTAAAGACCTC	CCACCGATCCACACAGAGTAC

### Western blotting

In vitro, BV2 cells were harvested at the indicated days and lysed in ice-old TNE buffer (150 mM NaCl; 10 mM Tris, pH 8.0; 1 mM EDTA; 10% glycerol; 1% NP-40 with phosphatase protease inhibitors). Lysates were centrifuged at 14,000×*g* at 4 °C for 10 min. Protein lysates were boiled in sample buffer and resolved on 10% SDS-PAGE gels. The following primary antibodies were used in this study: rabbit anti-iNOS (Abcam, ab15323, 1:100), rabbit anti-MCT1 (Millipore, AB3540P, 1:1000), mouse anti-MCT4 (Santa Cruz, sc-376140, 1:200), rabbit anti-PFKFB3 (Proteintech, 13763-1-AP, 1:2000), rabbit anti-STAT1 (Bioworld, BS1333, 1:500), rabbit anti-phospho-STAT1 (CST, 9167, 1:1000), and mouse anti-β-actin (Proteintech, 60008-1-Ig, 1:2000). The appropriate HRP-conjugated second antibodies (1:5000) were from Calbiochem. The blots were reacted with an ECL chemiluminescence system (Millipore) and the gray intensity analysis was performed using the Quantity One software.

### Immunofluorescence staining

Mice were anesthetized with 5% chloral-hydrate and perfused with 4% paraformaldehyde in PBS. Brains were removed and infiltrated with 30% sucrose for dehydration. Then brains were coated with OCT compound and sliced (40 μm). Slices were blocked in 5% normal goat serum in PBS for 1 h and incubated with primary antibodies against rabbit anti-Iba1 (Wako, 019-19741, 1:1000) or rabbit anti-CD86 (Abcam, ab53004, 1:200) overnight at 4 °C. After washing in PBS for 4 times, slices were incubated with anti-rabbit Alexa Fluor 488 or anti-rabbit Alexa Fluor 594 (Invitrogen) for 1 h at room temperature. Images were photographed using fluorescent microscopy (Nikon 80i) and Imaging computer program (NIS-Elements BR, Nikon). Finally, photographs were analyzed using the NIH Image J software.

### Lentivirus-mediated overexpression and RNA interference

BV2 cells were infected with lentivirus for 24 h before treatment with lipopolysaccharide (LPS, 1 μg/ml, Sigma) or recombinant mouse IL-4 (20 ng/ml, Proteintech). The siRNA sequences used for lentivirus were as followed: MCT1 siRNA: 5′-GCA ACG ACC AGT GAA GTA T-3 ′; MCT2 siRNA: 5′-GGA AGA GAG AGA AGG CAA A-3′; MCT4 siRNA: 5′-GGT CTT TGT GGT GAG CTA T-3′. The RNA interference plasmids used to package lentivirus were purchased from Thermo Scientific Open Biosysterms. The pUltra vector was used to package PFKFB3-overexpression lentivirus.

### Extracellular acidification rate (ECAR) assay

For real-time analysis of glycolysis, XFe 96 Extracellular Flux Analyzer (Seahorse Bioscience, North Billerica, MA, USA) was used to monitor ECAR in BV2 microglial cells as described previously [20]. In brief, BV2 cells infected with lentivirus were plated onto a Seahorse XFe 96-well cell culture microplate (3 × 10^4^ cells per well). After sedimentation for 1 h, BV2 cells were stimulated with LPS or vehicle and incubated at 37 °C in 5% CO_2_ for 24 h. Then culture medium was removed, and each well was washed two times with bicarbonate-free glycolysis stress media containing glutamine (pH 7.4). The cells were cultured at 37 °C without CO_2_ for 1 h. For ECAR measurement, the BV2 cells were monitored under basal conditions and in response to glucose (10 mM), oligomycin (2 μM), and 2-deoxyglucose (2-DG) (50 mM). The data obtained were normalized with cell number counts. Glycolysis values meant (maximum rate measurement before oligomycin injection) − (last rate measurement before glucose injection) while glycolytic capacity values represented (maximum rate measurement after oligomycin injection) − (last rate measurement before glucose injection).

### Cell viability test

The cell viability of microglia was determined by the 3-(4,5-dimethylthiazol-2-yl)-2, 5-diphenyltetrazolium bromide (MTT) assay. The cells were seeded in the 96-well plates and treated as indicated. MTT solution was added to each well, and incubated for 3 h at 37 °C under 5% CO_2_ atmosphere. The supernatant media were removed and dimethyl sulfoxide (DMSO) was used to dissolve the formazan crystals. Absorbance of the soluble formazan dye was measured at 490 nm with a microplate reader.

### Surgery, microinjection and open-field behavior test

After anesthetized with 5% chloral-hydrate, the mice were implanted a guide cannulas to the lateral ventricle. The coordinates were as follows: anteroposterior (AP) − 0.6 mm; lateral (L), +1.5 mm; dorsoventral (V), − 1.9 mm [21]. A stylus was placed into the guide cannula to avoid clogging. After recovery for 1 week, the mice were injected intraperitoneally with LPS (1 mg/kg). The microinjection of lactate (dissolved in 0.1 M PBS, 100 mM, 2 μL) or vehicle (0.1 M PBS) was performed by an infusion cannula, which is connected to a microsyringe and a microinjection pump (KD Scientific). The concentration of lactate was used as described previously [22].

After treatment with LPS for 8 h, open field test were used to evaluate the behavioral activity of mice [23]. The apparatus consists of a 40-cm × 40-cm black box and a camera at the top. Behavioral activity was recorded for 10 min after mice were placed in center of the field. A video tracking system (Smart) was used to score the total distance and resting time of each animal [24, 25]. The rearing activity was monitored by the camera. The mice were sacrificed after open field assay, and the brains were removed and used for immunofluorescence staining and RNA isolation.

### Statistical analysis

The SPSS 19.0 statistical program was used for data analysis. Statistical comparisons were performed by Student’s *t* test or ANOVA, followed by the LSD or Dunnett’s T3 post hoc test. *p* < 0.05 was considered significant. Values were presented as the mean ± SEM.

## Results

### MCT1, MCT4, and PFKFB3 were increased after LPS stimulation but not IL-4 in both BV2 microglia cell line and primary microglia

**Fig. 1:**
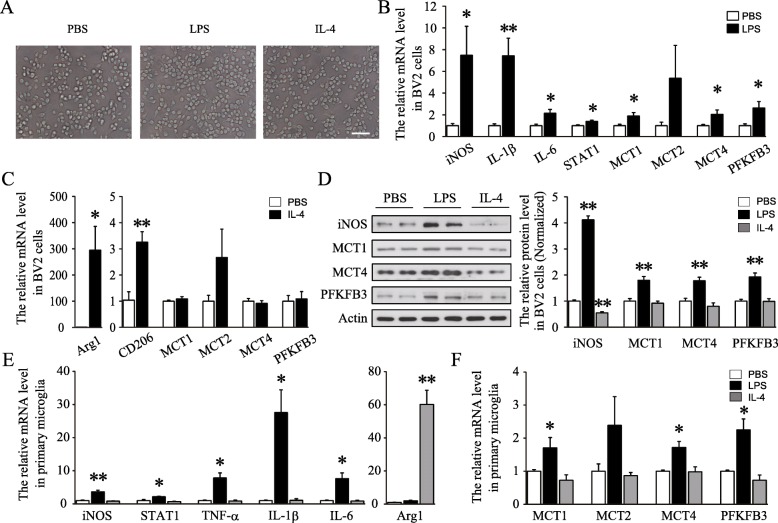
LPS-induced special increase of MCT1, MCT4, and PFKFB3 in BV2 cell line and primary microglia. **a** Representative morphology of BV2 cells after treatment with PBS, LPS, or IL-4. Scale bar = 200 μm. **b** Quantification of the mRNA levels of classical microglial markers and glycolysis-related regulatory factors after treatment with LPS for 24 h (*n* = 6–8 per group and errors represent S.E.M; *t* test). **c** Quantification of the mRNA levels of alternative M2 markers and glycolysis-related regulatory factors after treatment of IL-4 for 24 h (*n* = 5 per group; *t* test). **d** Representative immunoblots and relative quantification of iNOS, MCT1, MCT4, and PFKFB3 after treatment with PBS, LPS, or IL-4 (*n* = 6 per group; one-way ANOVA). **e**, **f** Quantification of the mRNA levels of these genes in primary cultured microglia in each group (*n* = 6 per group one-way ANOVA). *p < 0.05, **p < 0.01 versus PBS-treated group

Accumulated data showed the increase of glycolysis in IFNγ/LPS-induced classical M1 microglia [10, 16]. To validate the effect of glycolysis-related regulators such as MCTs and PFKFB3 on microglia polarization, BV2 microglia cell line was stimulated with LPS or IL-4, respectively. As previously reported, the morphology of LPS-treated BV2 cells showed large soma and fewer branches than control cells (Fig. 1a). Quantitative RT-PCR further verified the classical M1 and alternative M2 phenotype of BV2 cells after LPS or IL-4 stimulation respectively. LPS specially increased the mRNA levels of classical microglia markers, including iNOS, IL-1β, IL-6, and STAT1, while IL-4 largely increased the expression of Arg1 and CD206, which were alternative phenotype markers (Fig. 1b, c). Importantly, LPS, but not IL-4, specially increased the levels of MCT1, MCT4, and PFKFB3 in BV2 cells (Fig. 1b–d). However, the expression of MCT2 was increased in both M1 and M2 microglia, although no significant difference was found.

As shown in Fig. 1e, the phenotypes of LPS- and IL-4-induced classical or alternative microglial activation were further confirmed in cultured primary microglia. Besides, compared with control group, LPS significantly enhanced the expression of MCT1, MCT4, and PFKFB3, while no significant change was found in IL-4-stimulated primary microglia (Fig. 1f). Furthermore, in vivo experiments also displayed that intraperitoneal injection of LPS induced expression of MCT1 and PFKFB3 in the hippocampus (Additional file 1: Figure S1). Collectively, the specific increase of MCT1, MCT4, and PFKFB3 in LPS-stimulated microglia suggested a close relationship between increase of glycolysis and activation of classical microglia.

### Knockdown of MCT1 suppressed LPS-stimulated microglial classical polarization and glycolysis rate

**Fig. 2:**
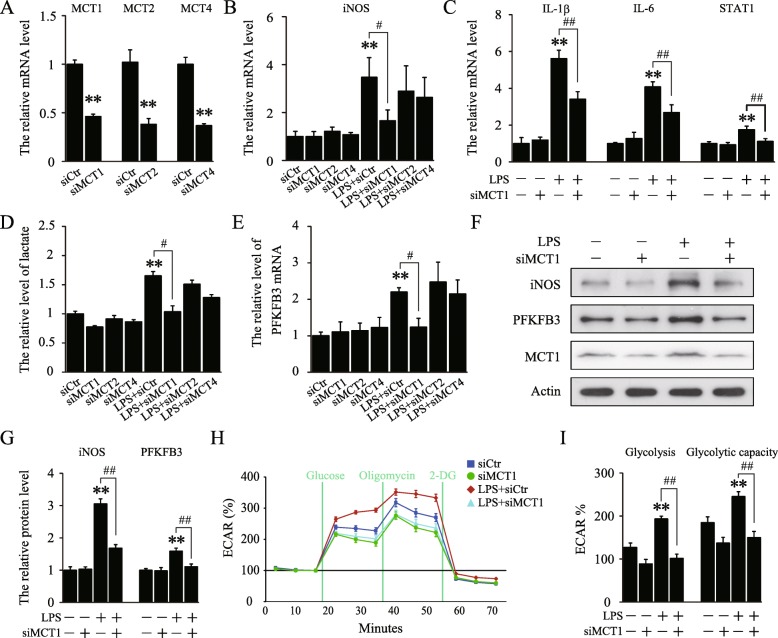
Knockdown of MCT1 inhibited LPS-stimulated classical microglial polarization and glycolysis rate in BV2 cells. **a** The interference efficiency of Lenti-siMCT1, Lenti-siMCT2, and Lenti-siMCT4, respectively, in BV2 cells (n = 6 per group and errors represent S.E.M; *t* test). **b** Quantification of the mRNA level of *iNOS* after treated with indicated lentivirus with or without LPS stimulation (*n* = 6–7 per group). **c** Quantification of the mRNA levels of *IL-1β*, *IL-6*, and *STAT1*, respectively, in each group (*n* = 5 per group). **d** Quantification of lactate levels in the cultured media in each group (*n* = 4 per group). **e** Quantification of the mRNA level of *PFKFB3* in each group (*n* = 5–6 per group). **f**, **g** Representative immunoblots and relative quantification of iNOS, PFKFB3, and MCT1 in each group (*n* = 6 per group). **h**, **i** The real-time extracellular acidification rate was measured by the sequential addition of glucose, oligomycin, and 2-DG in each group (*n* = 5 per group). *p and ^#^*p* < 0.05, **p and ^##^*p* < 0.01, two-way ANOVA followed by post hoc. Lenti-siCtr, Lenti-siMCT1, Lenti-siMCT2, and Lenti-siMCT4 are abbreviated as siCtr, siMCT1, siMCT2 and siMCT4, respectively

To further investigate the role of MCTs in classical polarization of microglia, lentiviruses expressing siRNA targeting *MCT1*, *MCT2*, or *MCT4* (Lenti-siMCT1, Lenti-siMCT2, and Lenti-siMCT4) and a control lentivirus with a scrambled sequence (Lenti-siCtr) were applied. Although each lentivirus effectively suppressed expression of corresponding MCT (Fig. 2a), only treatment with Lenti-siMCT1, but not Lenti-siMCT2 or Lenti-siMCT4, significantly downregulated LPS-induced expression of *iNOS* mRNA (Fig. 2b). Furthermore, compared with LPS-treated group, Lenti-siMCT1 reduced the mRNA levels of *IL-1β*, *IL-6*, and *STAT1*, suggesting the involvement of MCT1 in LPS-induced classical polarization of microglia (Fig. 2c).

To explore whether MCT1-mediated classical polarization of microglia is dependent on glycolysis, lactate in the cultured media was detected using the L-lactate assay kit. As shown in Fig. 2d, LPS treatment increased the production of lactate while knockdown of MCT1 reversed LPS-increased release of lactate. In accordance, Lenti-siMCT1 also significantly blocked LPS-induced expression of pro-glycolytic PFKFB3 enzyme (Fig. 2e–g). Further, ECAR assay showed that the glycolysis was enhanced in M1-polarized BV2 cells, displaying as increased ECAR value, while increased glycolysis was largely diminished after knockdown of MCT1 in LPS-stimulated BV2 cells (Fig. 2h, i). All above data suggested that MCT1 may promote classical microglial polarization via enhancement of glycolysis.

### MCT1 regulated LPS-stimulated classical microglial polarization via PFKFB3

**Fig. 3:**
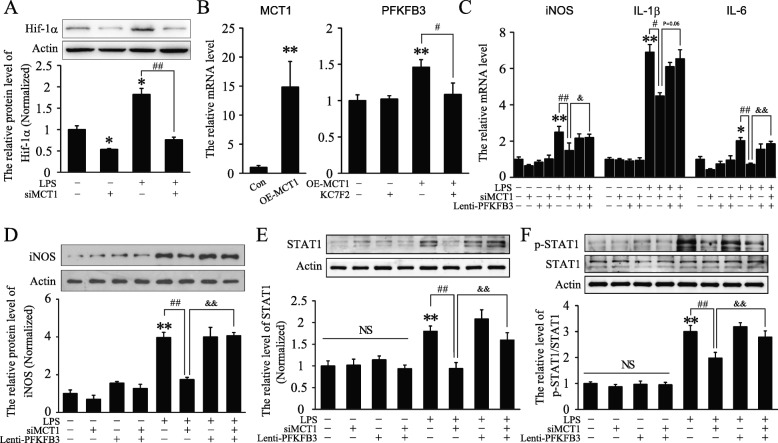
Overexpression of PFKFB3 rescued MCT1 silence-induced suppression of classical microglial polarization in BV2 cells. **a** Representative immunoblots and relative quantification of Hif-1α in each group (*n* = 4 per group and errors represent S.E.M). **b** The overexpression efficiency of Lenti-MCT1 and quantification of the mRNA level of *PFKFB3* in each group (*n* = 4 per group). KC7F2, an inhibitor of Hif-1α, 30 mM. **c**, Quantification of the mRNA level of *iNOS*, *IL-1β*, and *IL-6* after treatment with Lenti-siMCT1 and Lenti-PFKFB3 with or without LPS (*n* = 6–9 per group). **d**, **e** Representative immunoblots and relative quantification of iNOS and STAT1 protein, respectively, 24 h after LPS stimulation in each group (*n* = 6 per group). **f** Representative immunoblots and relative quantification of STAT1 and p-STAT1 after stimulation with LPS for 3 h in each group (*n* = 5–6 per group). *p, ^#^*p*, and ^&^*p* < 0.05, **p, ^##^*p*, and ^&&^*p* < 0.01, NS represents no significant difference; two-way ANOVA followed by post hoc

Hypoxia-inducible factor-1 (Hif-1) is a crucial transcription factor during classical microglial polarization [26, 27]. In this study, we found that the protein level of *Hif-1α* was increased after LPS stimulation and significantly decreased after knockdown of *MCT1* in BV2 cells (Fig 3a). Hif-1α is also regarded as an important regulator of glycolysis and its target genes include glucose transporter-1 (GLUT1) and PFKFB3 [27, 28]. KC7F2, a specific inhibitor of Hif-1α, was used to investigate the effect of Hif-1α in MCT1-regulated PFKFB3 expression. We found that treatment with KC7F2 reversed MCT1-increased expression of PFKFB3 in LPS-treated BV2 cells (Fig 3b), suggesting that MCT1 may regulate expression of PFKFB3 in a Hif-1α dependent manner. In addition, previous study has proved that knockdown of PFKFB3, the key enzyme in glycolysis, attenuated LPS-induced classical M1 polarization of macrophages [29]. Lentirivus-mediated MCT1 shRNA and lentivirus-mediated overexpression of PFKFB3 (Lenti-PFKFB3) were applied to address the role of PFKFB3 in MCT1-mediated classical microglial polarization. Results showed that, under LPS stimulation, overexpression of PFKFB3 significantly rescued the decreases of iNOS, IL-1β, and IL-6 mediated by Lenti-siMCT1 (Fig. 3c, d). However, either overexpression of PFKFB3 or knockdown of MCT1 showed no effect on the expression of Arg1 and CD206 (Additional file 1: Figure S2A, B). Besides, knockdown of MCT1 or overexpression of PFKFB3 had no effect on BV2 cell viability (Additional file 1: Figure S2C). Collectively, our data demonstrated the crucial role of PFKFB3 in MCT1-promoted classical polarization of microglia.

STAT1 has been reported to drive classical macrophage polarization and inflammatory gene expression [30–32]. Therefore, we addressed the effects of MCT1 on regulating STAT1 signaling. Consistently, compared with control group, MCT1 interference decreased LPS-induced expression of STAT1 protein, which was rescued by overexpression of PFKFB3 (Fig. 3e). However, as previous report [33], short stimulation of LPS (within 3 h) led to significant increase of p-STAT1 in cultured microglia although there was no significant change of total STAT1 protein. Moreover, compared with vehicle group, MCT1 knockdown inhibited LPS-induced phosphorylation of STAT1 which was blocked by PFKFB3 overexpression (Fig. 3f). All these results validated that MCT1 might promote classical microglial polarization via a pro-glycolysis manner.

### Lactate suppressed peripheral LPS-induced classical microglial polarization and neuroinflammation in mice

**Fig. 4:**
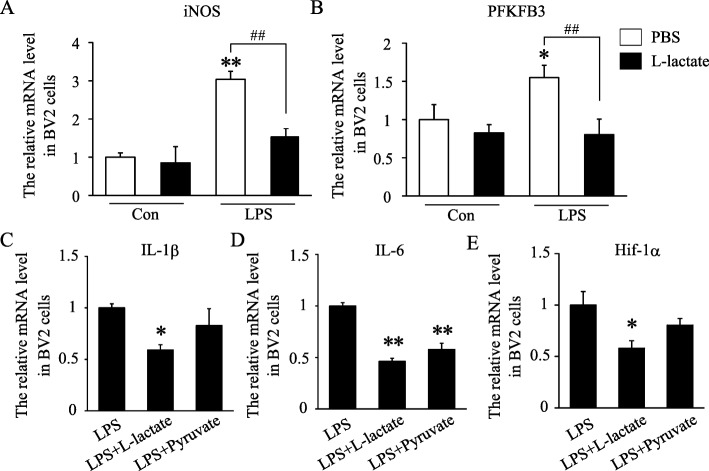
Lactate suppressed LPS-induced classical microglial polarization in BV2 cells. **a**, **b** Quantification of the mRNA levels of *iNOS* and *PFKFB3* in the treatment of lactate and LPS in BV2 cells (*n* = 9 per group and errors represent S.E.M). *p < 0.05, **p, and ^##^*p* < 0.01, two-way ANOVA followed by post hoc. **c**–**e** Quantification of the mRNA levels of *IL-1β*, *IL-6*, and *Hif-1α* in the treatment of lactate, pyruvate, and LPS in BV2 cells (*n* = 4–7 per group one-way ANOVA)

MCT1 is a transmembrane transporter of monocarboxylates and regarded as the key factor of anaerobic glycolysis to transport lactate into or outside of cell by facilitated diffusion [34]. Therefore, exogenous lactate stimulation may prevent MCT1-mediated excretion of lactate. Recently, lactate has been regarded as a substitute of glucose as a neuronal energy source to regulate neuronal activity and learning and memory [35]. Then the role of lactate on microglial function was assayed in this study. We found that exogenous lactate stimulation greatly suppressed the LPS-induced expression of iNOS and PFKFB3 in BV2 cells (Fig. 4a, b). Compared with stimulation of pyruvate, another substrate of MCT1, exogenous lactate seems to show larger effect on the decrease of IL-1β, IL-6, and Hif-1α (Fig. 4c–e). The above data suggested that lactate may mimic MCT1 interference effect on classical microglial polarization and neuroinflammation in vitro.

**Fig. 5:**
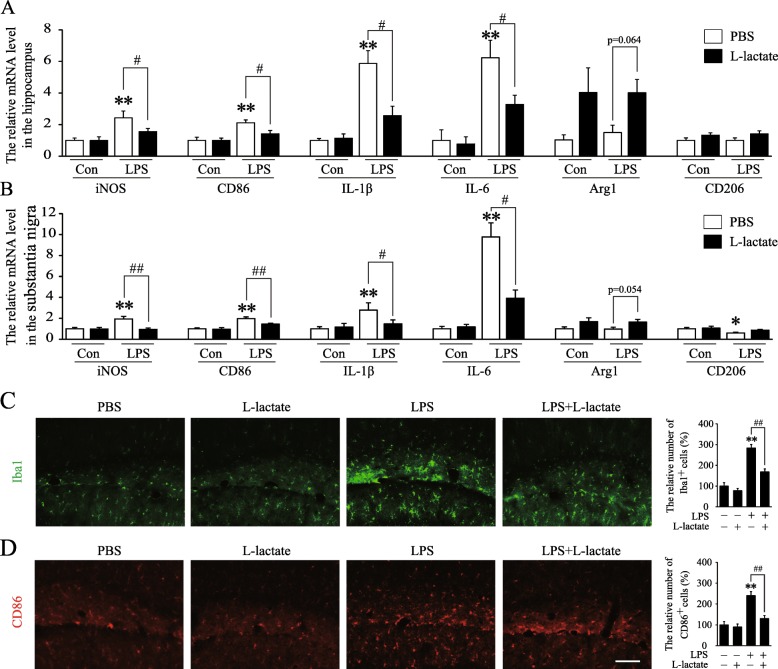
Intracerebroventricular injection of lactate reduced classical microglial polarization and neuroinflammation in the hippocampus and substantia nigra. **a**, **b** Quantification of the mRNA levels of microglial M1 and M2 markers in the hippocampus and substantia nigra, respectively, after intraperitoneal injection of LPS and intraventricular injection of L-lactate (*n* = 6–9 per group). **c**, **d** Immunostaining of classical microglia markers, CD86, and Iba1, and quantification of Iba1^+^ cells and CD86^+^ cells in the hippocampus in each group (*n* = 5–6 per group). *p and ^#^*p* < 0.05, **p and ^##^*p* < 0.01, two-way ANOVA followed by post hoc. Scale bar = 50 μm

To verify its role in vivo, lactate was injected intracerebroventricularly after peripheral administration with LPS. The substantia nigra consists of dopaminergic neuron and is an important brain region in regulation of locomotion. It has reported that the substantia nigra is susceptible to microglia-mediated inflammatory insult [25, 36]. The hippocampus plays critical roles in learning and memory, and neuroinlammatory processes in the hippocamous could lead to serious cognitive impairment [37]. In this study, we found that injection of LPS increased mRNA levels of pro-inflammatory markers in both the hippocampus and substantia nigra (Fig. 5a, b). Intracerebroventricular injection of lactate significantly reduced LPS-induced expression of M1-like phenotype markers, and also moderately enhanced the expression of Arg1 and CD206. In accordance with the quantitative RT-PCR data, the immunofluorescence results also showed that lactate significantly inhibited LPS increased the number of Iba1^+^ and CD86^+^ cells in the hippocampus (Fig. 5c, d) and substantia nigra (Additional file 1: Figure S3A,B). These data revealed that increased levels of lactate in the brain could reduce LPS-induced classical microglial polarization and neuroinflammation.

### Lactate ameliorated sickness behavior during a peripheral inflammatory challenge

**Fig. 6:**
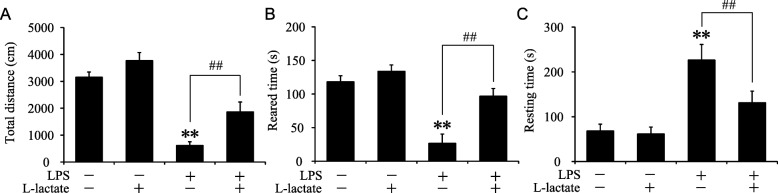
Intracerebroventricular injection of lactate ameliorated LPS-induced sickness behavior in mice. **a**–**c** Quantification of mice total distance, reared times, and resting times by open field tests after intraperitoneal injection of LPS and intraventricular injection of L-lactate (*n* = 8 per group and errors represent S.E.M, **p and ^##^*p* < 0.01; two-way ANOVA followed by post hoc)

As reported, stimulation of the peripheral innate immune system can induce activation of microglia and secretion of cytokines. Moderate cytokines act on neuron substrates, which is beneficial for the host. However, excessive production of cytokines by microglia induced neuroinflammation and sickness behavior [38]. It has been reported that systemic injection of LPS-induced inflammatory response can lead to sickness behavior [25]. According to the previous studies, sickness behavior may include anorexia, weight loss, and social withdrawal and so on [39]. Here, open field assay was used to investigate the effect of lactate on LPS-induced sickness behavior. As shown in Fig. 6, LPS decreased the total distance and reared time greatly. Interestingly, mice with intracerebroventricular injection of lactate showed significant improvement on total distance and reared times (Fig. 6a, b). In contrast, resting times were largely decreased after treatment with lactate compared with mice in LPS-treated group (Fig. 6c). In conclusion, these data demonstrated that intracerebroventricular administration of lactate could ameliorate LPS-induced neuroinflammation and sickness behavior.

## Discussion

Diversity and plasticity are hallmarks of cells of the monocyte-macrophage lineage. Microglia has been considered as the resident macrophages in the CNS. Due to the critical effect of activated microglia on neuroinflammation, reprogramming microglial polarization has become a potential therapy strategy in neurologic diseases [1, 3, 40, 41]. The current study provided several new insights on the glycolysis-mediated microglial polarization. First, we reported that MCT1, MCT4, and PFKFB3, the key glycolysis-related regulators, were significantly increased in classical microglia induced by LPS stimulation. Second, MCT1/PFKFB3-promoted glycolysis directed classical microglial polarization, which may rely on phosphorylation of STAT1. Third, exogenous lactate may mimic MCT1 interference effect on classical microglial polarization and neuroinflammation in vitro. Intracerebroventricular administration of lactate suppressed LPS-induced classical microglial polarization, accompanied by amelioration of neuroinflammation and sickness behavior in mice. Significantly, our research identified MCT1 as a new therapeutic target to attenuate neuroinflammation by reprogramming microglial polarization through metabolism.

Glucose has been regarded as the main energy source in the brain. Although emerging evidences show that glucose metabolism exists in neuron and astrocyte [35, 42], little is known about the effect of glucose metabolism on microglial function during pathological condition. In a rat middle cerebral artery occlusion model, enhanced expression of MCT1 and MCT2 has been detected in activated microglia [18]. However, there is no certain evidence to display the explicit effect of MCTs-mediated glycolysis on microglial polarization. Here, we showed that MCT1, MCT4, and PFKFB3 were increased in microglia after stimulation with LPS, while no changes were found in IL-4-treated cells. Moreover, knockdown of MCT1 significantly suppressed the expression of classical M1 markers, iNOS, IL-1β, IL-6, and STAT1, suggesting that MCT1 directly determine classical microglial polarization. Furthermore, knockdown of MCT1 reduced LPS-increased release of lactate and expression of pro-glycolytic PFKFB3 enzyme, and stimulation of additional lactate could mimic MCT1 interference effect on microglial activation. It is reported that MCT1 has a much greater affinity for lactate than MCT4. Besides, the expression of MCT1 and MCT4 in microglia may be different. The above data suggested that microglia may rely more on MCT1 than MCT4 to transport lactate [34]. The ECAR assay also revealed that knockdown of MCT1 directly decreased glycolytic rate in LPS-stimulated BV2 cells. These results confirm that MCT1-directed classical microglial polarization may be dependent on pro-glycolytic regulation.

STATs are well-established potent transcriptional factors in the regulation of cell growth and differentiation, apoptosis, and inflammation in immune cells, which can be activated after phosphorylation [33, 43]. STAT1 has been identified as an important regulator in macrophage and microglia M1 polarization and inflammatory gene expression. An impaired expression of IFN regulatory factor-1 and iNOS is detected in the macrophages derived from STAT1^-/-^ mice after LPS stimulation [31]. In microglia, a rapid increase of p-STAT1 was displayed within 3 h after LPS stimulation. Our results showed that knockdown of MCT1 significantly decreased the level of p-STAT1 after LPS stimulation. Therefore, we conclude that MCT1-mediated glycolysis may drive microglia to classical polarization through promoting phosphorylation of STAT1. However, the mechanism of MCT1 regulating STAT1 requires further investigation in the future.

**Fig. 7:**
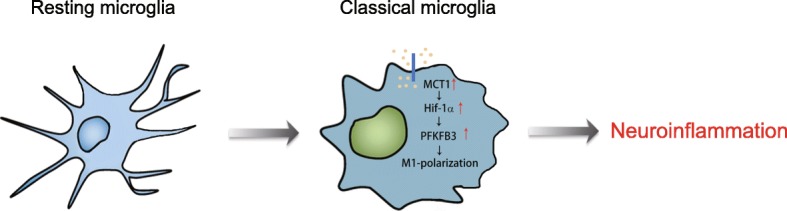
Schematic representation of MCT1/PFKFB3-mediated classical microglial activation and pro-inflammatory effect

Some metabolic enzymes and metabolites in the glycolysis and pentose phosphate pathway (PPP) have been reported to direct distinct polarization of macrophages [10]. PFKFB3 is an active ubiquitous PFK2 isoenzyme and a crucial regulator to promote glycolysis. Inhibition of glycolysis flux via knockdown of PFKFB3 largely hinders M1-polarization induced by LPS/IFN-γ [29]. Our results showed that both MCT1 deficiency and treatment with additional lactate decreased LPS-induced expression of PFKFB3 in microglia. By using a specific Hif-1α inhibitor, we found that MCT1 may regulate the expression of PFKFB3 via a Hif-1α dependent manner in microglia. In LPS-stimulated microglia, overexpression of PFKFB3 prevented knockdown of MCT1-impaired classical polarization. Here, we speculated that PFKFB3 might regulate the protein synthesis of markers of microglial polarization and phosphorylation of STAT1 through regulation of glycolysis and related metabolites. Above all, we conclude that MCT1 mediated classical microglial polarization through upregulation of PFKFB3 and further enhanced glycolysis flux. As MCTs are transporters of lactate, knockdown of MCT1 would directly prevent the transport of lactate from intracellular to outside, leading to lactate accumulation. The high accumulation of intracellular lactate may be a negative feedback regulation factor of glycolysis, which can inhibit the expression of Hif-1α. Alternatively, lactate, regarded as a signaling regulator, may also modulate expression of Hif-1α [44, 45]. As reported, PFKFB3 is one of Hif-1α target genes [28]. According to our results, we believed that MCT1 may enhance PFKFB3-mediated glycolysis via Hif-1α and finally promote microglial classical polarization (Fig. 7).

More importantly, our in vitro and in vivo experiments showed that treatment with lactate prevented microglia from classical polarization and attenuated neuroinflammation, which mimicked the effect of MCT1 interference. Evidences revealed that systemic administration of LPS was able to induce microglial M1 polarization and neuroinflammation, and finally led to sickness behavior [25]. Astonishingly, the increased levels of lactate in the brain attenuated LPS-induced sickness behavior. This result indicated that lactate may improve mice behavioral disorder through suppressing LPS-induced classical microglial polarization and neuroinflammation.

## Conclusions

In short, the present findings demonstrate that MCT1 enhances the expression of PFKFB3 via Hif-1α and thereby promotes classical microglial activation and pro-inflammatory effect. Exogenous lactate can mimic MCT1 interference effect on classical microglial polarization and neuroinflammation in vitro. Importantly, intracerebroventricular administration of lactate suppressed LPS-induced classical microglial polarization, accompanied by amelioration of neuroinflammation and sickness behavior in mice. Our data strongly suggested that attenuation of glycolysis flux targeting MCT1 in classical microglia maybe a potential therapy for brain diseases in the future.

## Supplementary information

**Additional file 1: Figure S1.** Intraperitoneal injection of LPS increased the expression of MCT1 and PFKFB3 in the hippocampus. A, Immunostaining of Iba1 (green) and MCT1 (Red) in the hippocampus in PBS- and LPS-treated groups (n = 4 per group). The white arrow represents Iba1 and MCT1-positive cells. B, Immunostaining of PFKFB3 in the hippocampus in each group (n = 4 per group). Scale bar = 50 μm. **Figure S2.** MCT1 and PFKFB3 have no effect on alternative microglial polarization and cell viability. A, The overexpression efficiency of Lenti-PFKFB3 in BV2 cells (n = 8 per group and errors represent S.E.M, **p < 0.01; *t* test). B, Quantification of the mRNA level of Arg1 and CD206 after treatment with Lenti-siMCT1 and Lenti-PFKFB3 under PBS and LPS-stimulated conditions (n = 8 per group and errors represent S.E.M). C, Knockdown of MCT1 or overexpression of PFKFB3 has no effect on BV2 cell viability (n = 5 per group and errors represent S.E.M). **Figure S3.** Intracerebroventricular injection of lactate reduced classical microglial polarization in the substantia nigra. A, B, Immunostaining of classical microglia markers, CD86 and Iba1, and quantification of Iba1^+^ cells and CD86^+^ cells in the hippocampus in each group (n = 5-6 per group). **p and ^##^*p* < 0.01, two-way ANOVA followed by post hoc. Scale bar = 50 μm.

## Data Availability

All data are available upon request.

## Abbreviations

MCT1: Monocarboxylate transporter 1
PFKFB3: 6-phosphofructo-2-kinase/fructose-2, 6-biphosphatase 3
IL-1β: Interleukin-1β
Hif-1α: Hypoxia-inducible factor-1α
CNS: Central nervous system
TNF-α: Tumor necrosis factor-α
ROS: Reactive oxygen species
TGF-β: Transforming growth factor-β
GLUT1: Glucose transporter 1
ECAR: Extracellular acidification rate
DMSO: Dimethyl sulfoxide
PPP: Pentose phosphate pathway
MTT: 3-(4,5-dimethylthiazol-2-yl)-2, 5-diphenyltetrazolium bromide
