# Supplementary material for: Monocarboxylate transporter 1 promotes classical microglial activation and pro-inflammatory effect via 6-phosphofructo-2-kinase/fructose-2, 6-biphosphatase 3
Source: J Neuroinflammation. 2019 Nov 28;16:240. doi: 10.1186/s12974-019-1648-4 (PMC6883695; doi:10.1186/s12974-019-1648-4)
Supplement: Supplementary file 1 — Additional file 1: Figure S1. Intraperitoneal injection of LPS increased the expression of MCT1 and PFKFB3 in the hippocampus. A, Immunostaining of Iba1 (green) and MCT1 (Red) in the hippocampus in PBS- and LPS-treated groups (n = 4 per group). The white arrow represents Iba1 and MCT1-positive cells. B, Immunostaining of PFKFB3 in the hippocampus in each group (n = 4 per group). Scale bar = 50 μm. Figure S2. MCT1 and PFKFB3 have no effect on alternative microglial polarization and cell viability. A, The overexpression efficiency of Lenti-PFKFB3 in BV2 cells (n = 8 per group and errors represent S.E.M, **p < 0.01; t test). B, Quantification of the mRNA level of Arg1 and CD206 after treatment with Lenti-siMCT1 and Lenti-PFKFB3 under PBS and LPS-stimulated conditions (n = 8 per group and errors represent S.E.M). C, Knockdown of MCT1 or overexpression of PFKFB3 has no effect on BV2 cell viability (n = 5 per group and errors represent S.E.M). Figure S3. Intracerebroventricular injection of lactate reduced classical microglial polarization in the substantia nigra. A, B, Immunostaining of classical microglia markers, CD86 and Iba1, and quantification of Iba1+ cells and CD86+ cells in the hippocampus in each group (n = 5-6 per group). **p and ##p < 0.01, two-way ANOVA followed by post hoc. Scale bar = 50 μm. [file 12974_2019_1648_MOESM1_ESM.doc]

Supplementary materials for

# Monocarboxylate transporter 1 promotes classical microglial activation and pro-inflammatory effect *via* 6-phosphofructo-2-kinase/fructose-2,6-biphosphatase 3

# Liang Kong^1^, Zehua Wang^1^, Xiaohong Liang^1^, Yue Wang^3^, Lifen Gao^1†^, Chunhong Ma^1,2,†^

1. Key Laboratory for Experimental Teratology of Ministry of Education and Department of Immunology, School of Basic Medical Sciences, Shandong University, Jinan, Shandong, 250012, P.R. China

# 2. Key Laboratory of Infection and Immunity of Shandong Province, School of Basic Medical Sciences, Shandong University, Jinan, Shandong, 250012, P.R. China

# 3. Department of Neurobiology, Shandong Provincial Key Laboratory of Mental Disorders, School of Basic Medical Sciences, Shandong University, Jinan, Shandong

# †Correspondence to Chunhong Ma or Lifen Gao: Ph. D, Key Laboratory for Experimental Teratology of Ministry of Education and Shandong Provincial Key Laboratory of Infection & Immunology, Department of Immunology, School of Basic Medical Sciences, Shandong University, 44 Wenhua Xi Road, Jinan, Shandong 250012, China. Tel/Fax: 86-531-88382038.

# E-mail: [**machunhong@sdu.edu.cn**](mailto:machunhong@sdu.edu.cn) or [**glfflg@sdu.edu.cn**](mailto:glfflg@sdu.edu.cn)


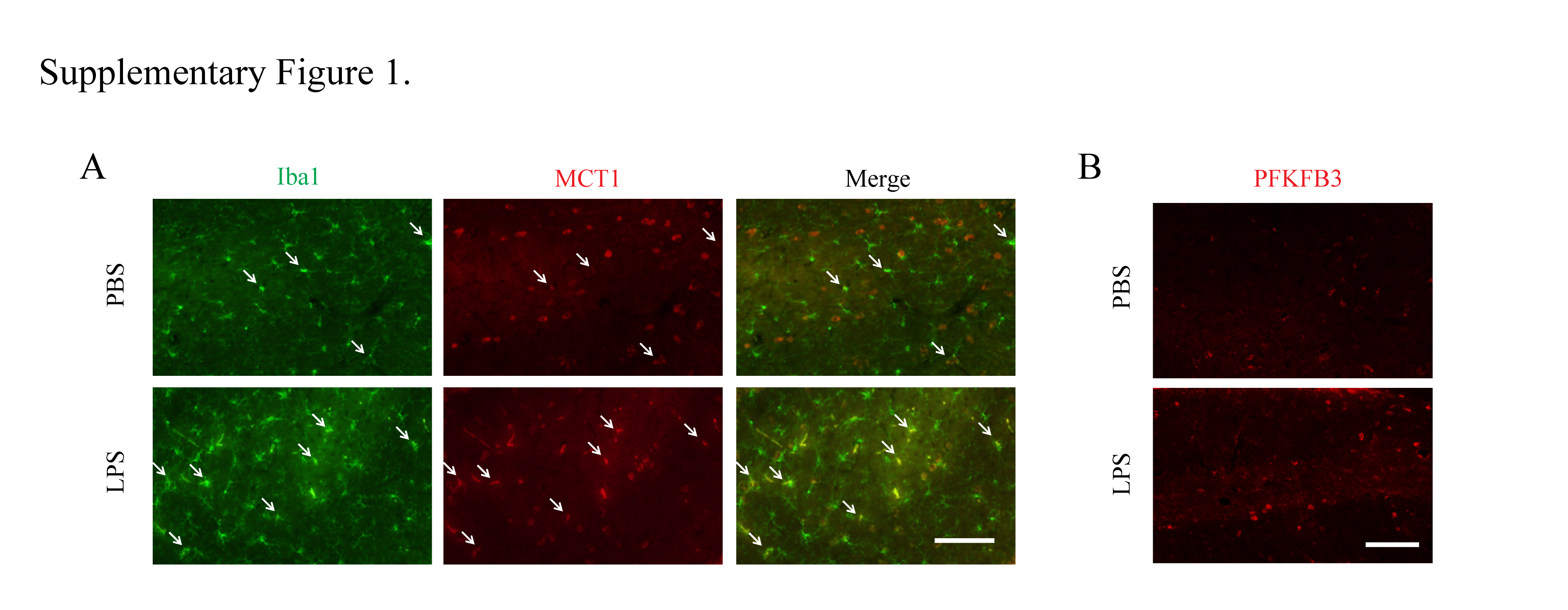


Supplementary Figure 1. Intraperitoneal injection of LPS increased the expression of MCT1 and PFKFB3 in the hippocampus. A, Immunostaining of Iba1 (green) and MCT1 (Red) in the hippocampus in PBS- and LPS-treated groups (n=4 per group). The white arrow represents Iba1 and MCT1-positive cells. B, Immunostaining of PFKFB3 in the hippocampus in each group (n=4 per group). Scale bar = 50 μm.


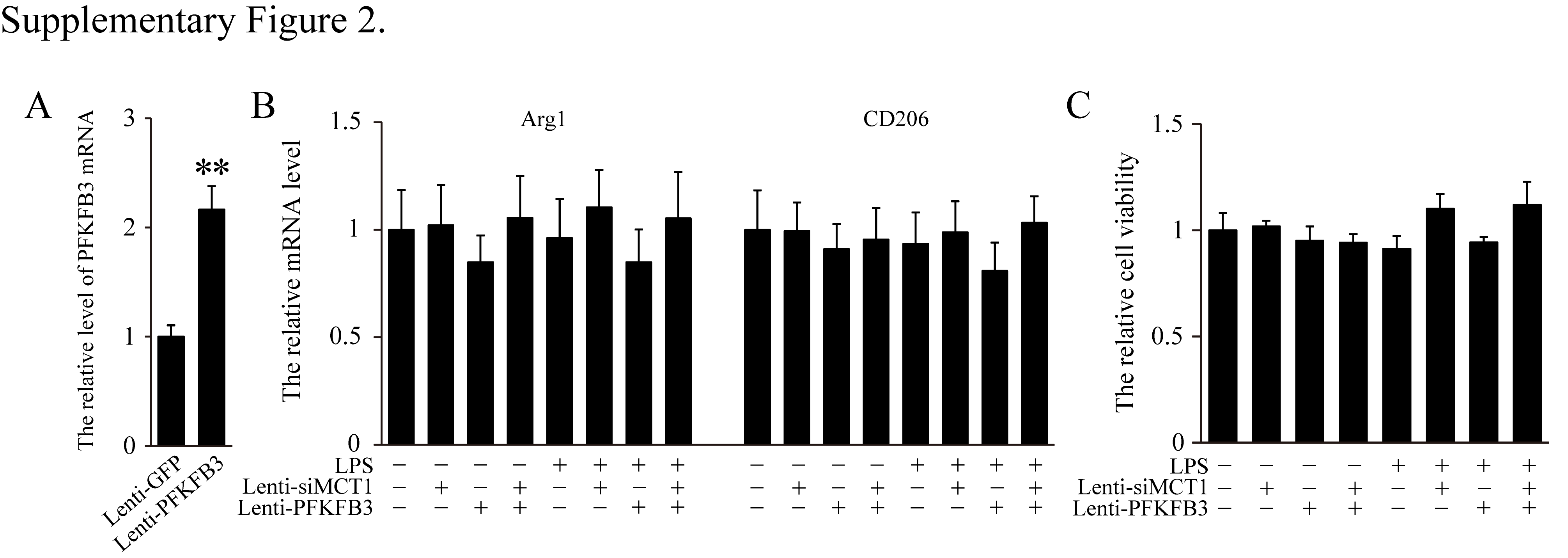


Supplementary Figure 2. MCT1 and PFKFB3 have no effect on alternative microglial polarization and cell viability. A, The overexpression efficiency of Lenti-PFKFB3 in BV2 cells (n = 8 per group and errors represent S.E.M, ***p* < 0.01; *t* test). B, Quantification of the mRNA level of Arg1 and CD206 after treatment with Lenti-siMCT1 and Lenti-PFKFB3 under PBS and LPS-stimulated conditions (n = 8 per group and errors represent S.E.M). C, Knockdown of MCT1 or overexpression of PFKFB3 has no effect on BV2 cell viability (n = 5 per group and errors represent S.E.M).


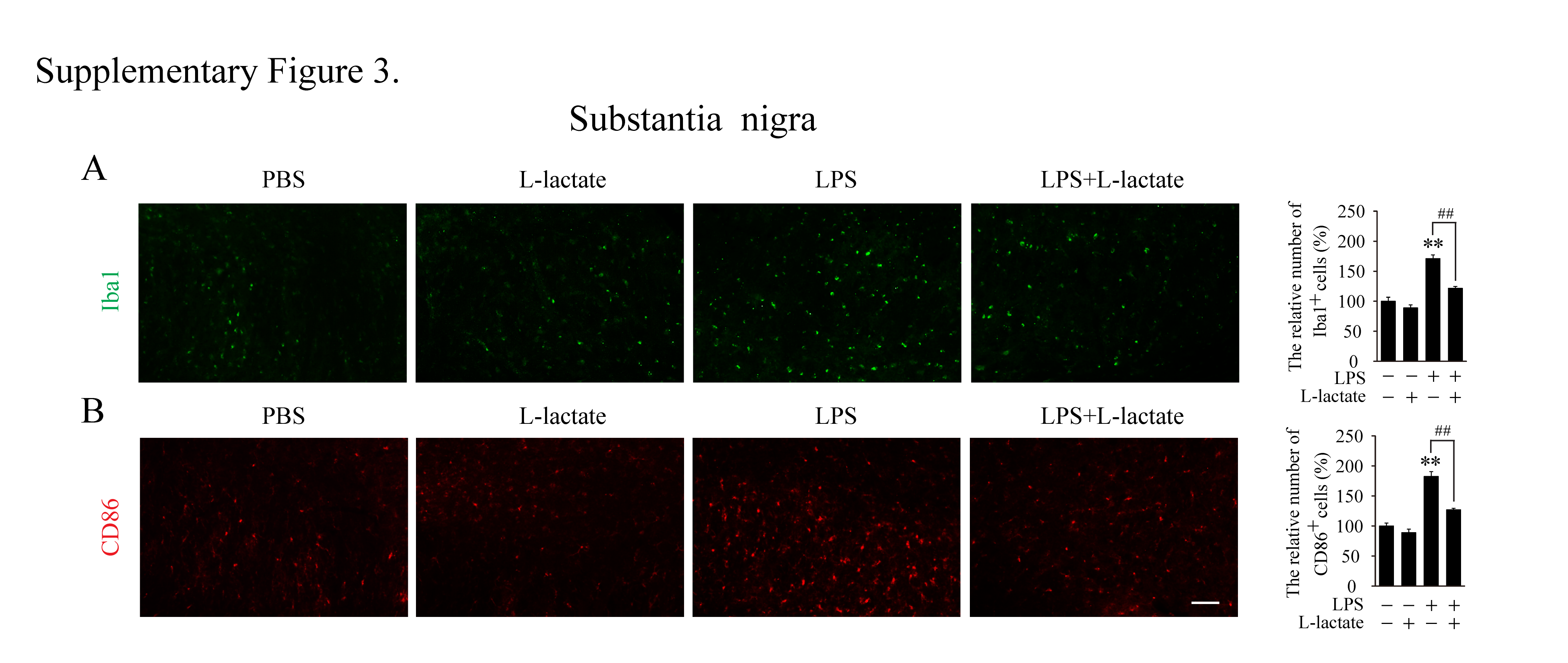


Supplementary Figure 3. Intracerebroventricular injection of lactate reduced classical microglial polarization in the substantia nigra. A, B, Immunostaining of classical microglia markers, CD86 and Iba1, and quantification of Iba1^+^ cells and CD86^+^ cells in the hippocampus in each group (n = 5-6 per group). ***p* and ^##^*p* < 0.01, two-way ANOVA followed by post hoc. Scale bar = 50 μm.
